# Ho! Every One to the Breeches!!

**Published:** 1866-08

**Authors:** 


### HO! EVERY ONE TO THE BREECHES!!

A little boy was chased by a cross dog. "Wern't you frightened' Charlie?" asked a friend.

"Well, no, Uncle; I wasn't scared, but I never wanted up a tree so badly in all my life," said Charlie.

Well, what of it? Why—

The "G. T. B." editor of the *Dental Times* isn't exactly frightened; but since the day that his mother spanked him he has not had such misgivings in reference to the encroachments of woman upon the rights of man. And the primary cause of this turmoil is a little girl out west who has gone and made a dentist of herself; and the finishing stroke is that she has been admitted to membership in a first class State Society; and, as if adding insult to injury, she has graduated in the Ohio College of Dental Surgery, and is, therefore, Lucy B. Hobbs, Doctor of Dental Surgery, which is something not provided for by the customs of society nor even laid down in the books.

But what is "G. T. B." going to do about it? That is the funniest of all. He tells us "I propose to offer an amendment to the constitution of the American Dental Association, at its next meeting in Boston, to allow none but males to be eligible as delegates from the local societies."

G(o) T(o), B(rother)! Let me expound unto you "a more excellent way." If Doctor Lucy should be at Boston as a delegate, before you offer your amendment, get her to fill a tooth for you, (if you have no decayed ones let her drill a hole in a sound one) and your objections to lady dentists will vanish into thin air. The doctor will do it well. The Ohio College will go her security; and it will appear so perfectly natural and proper to the patient that his amendment will never be thought of again. When there is but one lady graduate in the whole dental profession of the world, it looks like taking time by the fore-lock to shut her out.

The editor argues at length that woman is not fitted by nature for the practice of dentistry. I do not propose to notice his arguments; they are average specimens of special pleading. Their main point seems to be that, as the practice occupies both mind and body it is too hard for women. The fallacy of this will appear to any one who thinks of what is ordinarily required of and accomplished by women. Filling a tooth requires less effort of mind and body than getting up a dinner; and the whole of dental practice is mere play, both of mind and body, when compared with house-keeping.

Dentists are too liable to act the fool by attempting to magnify their calling. They talk eloquently about its cares and perplexities, when, as compared with medicine or theology, it has none. They prate about its difficulties as an excuse for not overcoming them, and they groan their Jeremiads over its toils and fatigues, to prove that women and weaklings should be kindly kept from its pale. The truth is, dentistry is a nice, clean, cosy, comfortable, easy profession, as compared with others. Its labor is easy, its studies are pleasant pastime, its offices are airy parlors; its hours are regular; its patrons are intelligent, good company; its members are jolly, pleasant companions; its remunerations are reasonable, its responsibilities are light, its trials are few, its vexations are not worth mentioning; its temptations are slight, its position is honorable and honored, and its progress toward perfection is rapid. It is a profession fit for strong men or weak men, for old men or young men, for women and for girls; fit, always fit for the competent and the honest, but never fit for the unprincipled and the incompetent. And the man who whines about its cares and its burdens, its toils and its trials, is unworthy of it and a discredit to it. W.

